# Gender‐related difference in altered fractional amplitude of low‐frequency fluctuations after electroacupuncture on primary insomnia patients: A resting‐state fMRI study

**DOI:** 10.1002/brb3.1927

**Published:** 2020-11-04

**Authors:** Xiao‐Hua Shi, Yu‐Kai Wang, Tie Li, Hong‐Yu Liu, Xin‐Tong Wang, Zhi‐Hong Wang, Jing Mang, Zhong‐Xin Xu

**Affiliations:** ^1^ Department of Neurology China‐Japan Union Hospital of Jilin University Changchun China; ^2^ Department of Acupuncture and Moxibustion Changchun University of Chinese Medicine Changchun China

**Keywords:** amplitude of low‐frequency fluctuations, electroacupuncture, functional magnetic resonance imaging, primary insomnia

## Abstract

**Background:**

Primary insomnia (PI) is defined as a sleep disorder with no definite cause or inducement. Electroacupuncture, a treatment of inserting needles into specific points on the body surface and applying electrical stimulation, has been proved effective in treating PI with minimal adverse effects. However, the influence of gender difference on the clinical treatment efficacy of electroacupuncture for PI patients remains unclear. Therefore, we designed a clinical trial to compare the clinical treatment efficacy of electroacupuncture for PI patients with different genders. The research on the mechanism of electroacupuncture suggested it could modulate the sleep and wakefulness by activating or deactivating brain regions via a needling/tactile somatosensory specific stimulus. Therefore, we also designed a resting‐state functional magnetic resonance imaging (rs‐fMRI) study to detect the spontaneous brain activity of PI patients before and after the electroacupuncture treatment.

**Method:**

Thirty PI patients were recruited to accept 5‐week electroacupuncture treatment on HT‐7. Athens Insomnia Scale (AIS) and Pittsburgh sleep quality index (PSQI) questionnaires were used to evaluate the clinical treatment efficacy. Rs‐fMRI was employed to observe the spontaneous brain activity in the resting state at the baseline and after 5 weeks of electroacupuncture treatment, which was measured by the fractional amplitude of low‐frequency fluctuations (fALFF).

**Result:**

The AIS and PSQI scores were significantly decreased both in the female PI group and the male PI group after treatment. The decreased PSQI of female patients was significantly more than that of male patients (*p* < .05). The gender‐related difference in the cerebral response to electroacupuncture was mainly in posterior cingulate and supramarginal gyrus.

**Conclusion:**

There is a gender‐related difference in the clinical treatment efficacy of electroacupuncture for PI patients, and female patients may benefit more from electroacupuncture. Gender‐related differences in the cerebral response to electroacupuncture may be one of the factors affecting clinical treatment efficacy.

## Graphical abstract

The brain regions of primary insomnia patients responding to acupuncture were mainly related to emotional and cognitive function. And the gender‐related difference of cerebral response to acupuncture could affect the clinical efficiency.

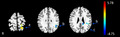

## 1 INTRODUCTION

Primary insomnia (PI), which has an increasing incidence, is a globally prevalent sleep disorder characterized by the difficulty in initiating sleep or maintaining sleep (Buysse, 2013; Irwin, 2015). In general, treatments for PI include nonpharmacologic and pharmacologic therapies. Cognitive‐behavioral therapies, the recommended nonpharmacologic therapies for insomnia, are still limited in clinical use due to the lack of qualified psychologists with sufficient time and knowledge (Riemann et al., 2017; van der Zweerde et al., 2016). Pharmacological therapies are also limited because of potential risks and adverse side effects (Lai et al., 2014). Therefore, the exploration of a more convenient treatment with fewer side effects for PI patients has lasted for more than a decade.

Acupuncture, a technique of inserting needles into particular body points, has been proven to be effective and show minor side effects in the treatment of insomnia (Yin et al., 2017). It has been increasingly used in the United States as a treatment for insomnia (Wang et al., 2018). Electroacupuncture (EA) is a modified method of conventional acupuncture, which supplies a sequential physical electrical stimulation by inserting acupuncture needles connected to a microcurrent stimulator. Since the body tissue is a kind of electrical conductor, it is believed that the electrical impulses can reinforce the stimulation through the needles at acupoints. Several reported studies have also proved the efficacy of EA in treating insomnia (Li et al., 2018).

Primary insomnia is generally considered to be a disorder of hyper‐arousal in the physiologic, emotional, or cognitive network (Levenson et al., 2015; Riemann et al., 2010). Previous studies have demonstrated aberrant regional spontaneous brain activity in sleep disorders as well as gender differences in these brain areas (Dai et al., 2012, 2016). EA can modulate brain activity via a needling/ tactile somatosensory specific stimulus, which may regulate the sleep and wakefulness (Napadow et al., 2012). However, studies have identified gender as a factor influencing the clinical treatment efficacy of some treatments for insomnia (Nowakowski and Meers 2019). We would like to know whether there is a gender‐related difference in the treatment efficacy of EA for PI and the possible causes for it. Therefore, a clinical trial and a resting‐state functional magnetic resonance imaging (rs‐fMRI) study were designed to compare the treatment efficacy of EA for PI patients with different genders and to observe the spontaneous brain activity of PI patients before and after the EA treatment.

Rs‐fMRI is considered as a measure of blood oxygenation level–dependent (BOLD) signals in brain tissue in the resting state (Chen & Glover, 2015). The amplitude of low‐frequency fluctuations (ALFF) can directly demonstrate the BOLD signal and reflect spontaneous fluctuations in the voxels under the resting state (Fransson, 2005). The simple calculation and reliable characterization (Zuo et al., 2010) of the ALFF measurement make it a useful tool to investigate the spontaneous brain activity. A modified calculation called the fractional amplitude of low‐frequency fluctuation (fALFF) refers to the ratio of the power spectrum of low frequency (0.01–0.08 Hz) to that of the entire frequency range. Its role in suppressing nonspecific noise components and improving the effectiveness in exploring local BOLD signals has been proved (Zou et al., 2008). In this trial, we compared the clinical treatment efficacy of EA for PI patients with different genders and observed the fALFF in patients with different genders before and after treatment, in order to explore the possible reasons for the gender‐related difference in the treatment efficacy of EA.

## 2 METHOD

### 2.1 Participants

From September 2017 to September 2018, thirty PI patients from the outpatient clinic in the Neurological Department of China‐Japan Union Hospital of Jilin University and the Neurological Department of Changchun University of Chinese Medicine were recruited in this study (No. NCT02448602, registered on 14/04/2015). A written signed informed consent was obtained from each participant. All PI patients should satisfy the following criteria.

Inclusion criteria: (a) patients aged from 18 to 65 years old; (b) patients with sleep onset latency or wake after sleep onset of >30 min at least 3 nights per week, with symptoms lasting for ≥3 months; (c) patients with a Pittsburgh sleep quality index (PSQI) score of >7 and Athens Insomnia Scale (AIS) score of ≥6.

Exclusion criteria: (a) patients with uncontrolled medical or psychiatric conditions; (b) patients with the self‐rating anxiety scale (SAS) or self‐rating depression scale (SDS) score of ≥50; (c) patients diagnosed with comorbid sleep disorders, such as obstructive sleep apnea; (d) patients with alcohol and/or other drug abuse or dependence; (e) patients who received hypnotic or sedating medications or accepted electroacupuncture treatment in the recent 1 month.

### 2.2 Procedures of the electroacupuncture

**FIGURE 1:**
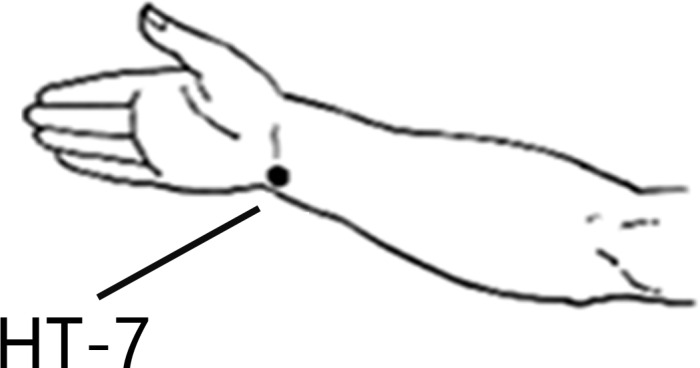
The location of acupoint HT‐7

Primary insomnia patients accepted EA treatment for 5 weeks on HT‐7, an acupoint proved effective and widely used in clinical treatment (Zhao, 2013; Figure 1). During the EA stimulation, a 25 × 0.35 mm sterile and reusable acupuncture silver needle was inserted into the bilateral acupoints at a depth of 15–20 mm. Once the De‐Qi sensation (Takamoto et al., 2010) was elicited, the handle of the needle was connected to an EA machine (Suzhou Medical Appliance Factory, China) with a frequency of 15 Hz and an intensity of 1 (9 V, ≤10 mA). The Massachusetts General Hospital acupuncture sensation scale (MASS) was adopted to rate the De‐Qi sensations during the EA. Sensations, including soreness, numbness, heaviness, warmth, coldness, sharp pain, and dull pain, were scored to screen patients with the De‐Qi sensation. The EA treatment lasted for 5 weeks, 30 min each time and five times per week. The administration of treatment and the operation of EA were performed by a professional acupuncturist, who had engaged in clinical acupuncture for more than 3 years.

### 2.3 Observation and analysis of clinical treatment efficacy

Primary insomnia patients accepted the AIS and PSQI questionnaires at the baseline and at the end of the 5‐week treatment to evaluate the treatment efficacy. The AIS questionnaire is a self‐administered psychometric instrument consisting of eight items. Each item of the AIS can be rated from 0 to 3 for a total score range of 0–24, with a score of “0” indicating no problem at all and a score of “24” indicating very serious problems in all areas (Soldatos et al., 2000). The PSQI questionnaire consists of 19 self‐rated questions, which are grouped into seven component scores ranging from 0 to 3 each. The seven component scores are then summed to yield a global PSQI score, which has a range of 0–21, with higher scores indicating worse sleep quality (Buysse et al., 1989).

The clinical data, including age, gender, AIS, PSQI, SAS, and SDS, of female PI patients (FPIs) and male PI patients (MPIs) were analyzed with SPSS 18.0 statistical software. Firstly, the AIS/PSQI scores of FPIs and MPIs at the baseline were analyzed through normality analysis. Then, age, gender, SAS, and SDS of FPIs and MPIs were analyzed by an independent‐sample *t* test. The AIS/PSQI scores of FPIs and MPIs at the baseline and after the treatment were analyzed by a two‐factorial ANOVA test. The change of AIS/PSQI scores between the two groups was analyzed by an independent‐sample *t* test. All data were expressed as mean ± standard deviation.

### 2.4 Rs‐fMRI data acquisition

Primary insomnia patients all received an rs‐fMRI assessment from 8:00 a.m. to 10:00 a.m. in an awake state. The fMRI scan was completed on a 3.0 T whole‐body MRI scanner (MAGNETOM‐skyra‐SIEMENTS). The MRI sequences are detailed as (a) T1‐weighted MRI: Data were acquired by a magnetization‐prepared rapid gradient‐echo sequence with 192 continuous sagittal slices that covered the whole brain, with TR/TE at 700 ms/11 ms, FOV at 256 × 256 mm, and a voxel size of 1 × 1; (b) rs‐fMRI: Data were acquired by an echo‐planar imaging sequence sensitive to BOLD contrast with 36 slices that covered the whole brain, with TR/TE/FA at 2,020 ms/30 ms/90°, FOV at 106 × 106 mm, and a voxel size of 2.4 × 2.4. The rs‐fMRI scan lasted 200 TR.

### 2.5 Rs‐fMRI data processing

Rs‐fMRI data were preprocessed by the Data Processing Assistant for Resting‐State fMRI (DPARSF, http://rfmri.org/DPARSF) package (Yan & Zang, 2010) and analyzed with Statistical Parametric Mapping toolbox (SPM8, Welcome Department of Imaging Neuroscience, Institute of Neurology, London; http://www.fil.ion.ucl.ac.uk/spm). Data of Digital Imaging and Communications in Medicine (DICOM) were converted into NIFTI data. The first 10 images of each functional time series were discarded; while all slices of the remaining images were processed by slice‐timing adjustment and realigned to the middle volume. Then, the time series of images were motion‐corrected. The dataset in which the translation or rotation parameters exceeded 1.5 mm or 1.5 degrees of the rotation was discarded. Then, the realigned functional images were spatially normalized to the Montreal Neurological Institute (MNI) space through the normalization parameters estimated by the T1 structural image unified segmentation. Next, they were resampled to a resolution of 3 × 3 × 3 mm^3^ voxels. Afterward, the normalized data were spatially smoothed by a 6 mm full‐width half‐maximum Gaussian kernel. Linear detrending and nuisance linear regression (including the white matter, the cerebrospinal fluid and head motion parameters) were performed, and a temporal bandpass filter (0.01–0.08 Hz) was employed to reduce the effects of head motion and nonneuronal BOLD fluctuations (He et al., 2007). ALFF and fALFF were calculated with DPARSF package for each subject (Zou et al., 2008).

### 2.6 Rs‐fMRI data analysis

The gender‐related difference of fALFF between FPIs and MPIs was analyzed by regression analysis with gender as a variable (an uncorrected voxel‐wise *p * <  .001 and cluster‐level FWE correction *p *<  .05, cluster size > 30).

The altered fALFF of MPIs and the altered fALFF of FPIs after treatment (an uncorrected voxel‐wise *p * <  .001 and cluster‐level FWE correction *p *<  .05, cluster size > 30), and the difference of altered fALFF between MPIs and FPIs were analyzed by one‐way ANOVA within‐subjects test (Flexible Factorial Model) (an uncorrected voxel‐wise *p * <  .005 and cluster‐level FWE correction *p *<  .05, cluster size > 30). The results were presented by REST 1.8v software (Song et al., 2011).

### 2.7 Patient safety

Any adverse events related to the EA treatment, including unfavorable or unintended signs, symptoms, or diseases occurring after treatment, were observed and reported. The patients would immediately terminate the trial if an adverse event happened.

### 2.8 Ethical statement

The experiment was conducted in accordance with the ethical guidelines of the Declaration of Helsinki, all methodologies were approved by the Ethics Committee of Changchun University of Chinese Medicine (Reference: CCZYFYLL2014‐043). A written signed informed consent was provided by each participant.

## 3 RESULTS

### 3.1 Clinical treatment efficacy

In our study, 12 male PI patients (45.00 ± 12.70 years old) and 18 female PI patients (52.88 ± 4.55 years old) were recruited. The AIS (*p* = .133) and PSQI (*p* = .101) scores of PI patients conformed to normal distribution. There were no significant differences in PSQI, AIS, SAS, or SDS scores between FPIs and MPIs at the baseline (*p* > .05). After the 5‐week EA treatment, the PSQI and AIS of FPIs and MPIs were both significantly decreased (*p* < .001). Furthermore, the decreased PSQI score of FPIs was significantly higher than that of MPIs (*p* = .02; Table 1).

**TABLE 1:** Demographic characteristic at baseline and after treatment in FPIs and MPIs

Parameter	FPIs	MPIs	*p* value
Gender			
Male		12	
Female	18		
Age (years)	52.88 ± 4.55	45.00 ± 12.70	.022
SAS	36.50 ± 1.98	37.67 ± 2.06	.136
SDS	37.00 ± 2.45	38.17 ± 1.75	.140
PSQI‐pre	15.00 ± 3.07	14.33 ± 1.56	.494
PSQI‐post	6.33 ± 1.08^a^	7.58 ± 1.08^a^	.005
PSQI‐decrease	8.67 ± 2.66	6.75 ± 0.87	.023
AIS‐pre	13.22 ± 3.66	12.17 ± 1.53	.286
AIS‐post	2.22 ± 0.81^b^	2.50 ± 0.80^b^	.362
AIS‐decrease	11.00 ± 3.66	9.67 ± 1.56	.245

Abbreviations: AIS, Athens Insomnia Scale; FPIs, female primary insomnia patients; MPIs, male primary insomnia patients; PAQI‐post, the value after the electroacupuncture treatment; PSQI, Pittsburgh sleep Quality index; PSQI‐decrease, the decrease between PSQI‐pre and PSQI‐post; PSQI‐pre, The value before the electroacupuncture treatment; SAS, self‐rating anxiety scale; SDS, self‐rating depression scale.

^a^ *P* < .001(comparing with the PSQI‐pre).

^b^ *P* < .001(comparing with the AIS‐pre).

### 3.2 The fALFF at the baseline and after electroacupuncture treatment

The difference between the fALFF of FPIs and the fALFF of MPIs mainly existed in the bilateral middle temporal gyrus, right cuneus, left supramarginal gyrus, left precuneus, left posterior cingulate, bilateral inferior parietal lobule, and bilateral cingulate gyrus. The fALFF values of these regions in MPIs were higher than those in FPIs (an uncorrected voxel‐wise *p * <  .001 and cluster‐level FWE correction *p *<  .05, cluster size > 30; Table 2).

**TABLE 2:** The difference between the fALFF of MPIs and the fALFF of FPIs at the baseline

Brain regions	BA	Side	Cluster size	MNI			*t*‐value
				*X*	*Y*	*Z*	
Middle temporal gyrus	37	L	39	−48	−51	−6	7.34
Middle temporal gyrus		R	45	60	−24	−9	6.45
Cuneus	18	R	79	6	−96	0	7.48
Supramarginal gyrus		L	63	−39	−54	27	6.45
Precuneus Posterior cingulate	31	L	153	−6	−66	27	6.24
Inferior parietal lobule	40	L	122	−51	−42	39	7.68
Cingulate gyrus		L	33	−6	−39	39	6.93
Cingulate gyrus		R	31	18	−27	45	7.19
Inferior parietal lobule Middle temporal gyrus	39	R	220	54	−57	42	6.84
Postcentral gyrus		L	49	−15	−45	63	8.63

Note

Anatomical locations, approximate Brodmann areas (BA), and Montreal Neurological Institute (MNI) coordinates, correspond to the *t*‐values of representative peaks within each cluster were reported. L: left. R: right. Positive *t*‐value suggested male patients had higher fALFF values in this area. All regions reached an uncorrected voxel‐wise *p*  <  .001 and cluster‐level FWE correction *p* <  .05, cluster size > 30.

**FIGURE 2:**
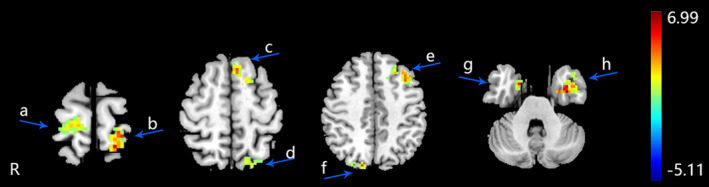
The altered fALFF regions of MPIs after 5 weeks electroacupuncture. (an uncorrected voxel‐wise *p*  <  .001 and cluster‐level FWE correction *p* <  .05, cluster size > 30). R: right brain. a: right postcentral gyrus, b: left postcentral gyrus, c: left superior frontal gyrus, d: left superior parietal lobule, e: left middle frontal gyrus, f: right precuneus, g: right parahippocampal gyrus, h: left middle temporal gyrus

**FIGURE 3:**
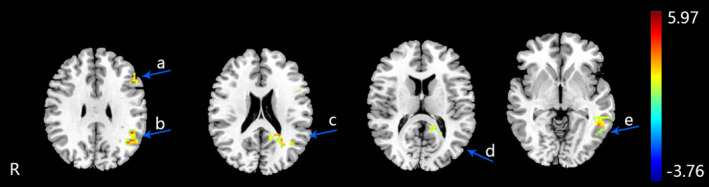
The altered fALFF regions of FPIs after 5 weeks electroacupuncture. (an uncorrected voxel‐wise *p*  <  .001 and cluster‐level FWE correction *p* <  .05, cluster size > 30). R: right brain. a: left inferior frontal gyrus, b: left supramarginal gyrus, c: left superior temporal gyrus, d: left posterior cingulate, e: left middle temporal gyrus

After the 5‐week EA treatment on HT‐7, the fALFF of MPIs increased in the left middle temporal gyrus, left superior frontal gyrus, left middle frontal gyrus, left medial frontal gyrus, right precuneus, right parahippocampal gyrus, left superior parietal lobule, and bilateral postcentral gyrus (an uncorrected voxel‐wise *p * <  .001 and cluster‐level FWE correction *p *<  .05, cluster size > 30) (Table 3 and Figure 2). The fALFF of FPIs increased in the left middle temporal gyrus, left posterior cingulate, left inferior frontal gyrus, left middle frontal gyrus, left superior temporal gyrus, and left supramarginal gyrus (an uncorrected voxel‐wise *p* < .001 and cluster‐level FWE correction *p *<  .05, cluster size > 30) (Table 4 and Figure 3).

**TABLE 3:** Altered fALFF signal of MPIs after 5‐weeks EA treatment at HT‐7

Brain regions	BA	Side	Cluster size	MNI			*t*‐value
				*X*	*Y*	*Z*	
Middle temporal gyrus	21	L	100	−36	−6	−33	6.90
Superior frontal gyrus	6	L	112	−6	21	60	5.59
Middle frontal gyrus	9	L	68	−33	15	39	5.94
Medial frontal gyrus		L	51	−3	60	9	4.68
Precuneus	19	R	57	15	−84	45	5.49
Parahippocampal gyrus	28	R	33	21	3	−33	5.79
Superior parietal lobule		L	36	−21	−63	54	4.52
Postcentral gyrus		L	44	−18	−48	75	6.26
Postcentral gyrus		R	44	18	−30	75	4.63

Note

Anatomical locations, approximate Brodmann areas (BA), and Montreal Neurological Institute (MNI) coordinates, correspond to the *t*‐values of representative peaks within each cluster were reported. L: left. R: right. A positive *t*‐value means that fALFF was increased in this brain region. All regions reached an uncorrected voxel‐wise *p*  <  .001 and cluster‐level FWE correction *p* <  .05, cluster size > 30.

**TABLE 4:** Altered fALFF signal of FPIs after 5‐weeks EA treatment at HT‐7

Brain regions	BA	Side	Cluster size	MNI			*t*‐value
				*X*	*Y*	*Z*	
Middle temporal gyrus	21	L	57	−51	−51	−3	4.79
Posterior cingulate		L	65	−18	−48	18	5.56
Inferior frontal gyrus Middle frontal gyrus		L	33	−51	12	24	4.63
Superior temporal gyrus	39	L	71	−42	−60	27	5.00
Supramarginal gyrus	39	L	73	−48	−48	30	5.17

Note

Anatomical locations, approximate Brodmann areas (BA), and Montreal Neurological Institute (MNI) coordinates, correspond to the *t*‐values of representative peaks within each cluster were reported. L: left. R: right. A positive *t*‐value means that fALFF was increased in this brain region. All regions reached an uncorrected voxel‐wise *p*  <  .001 and cluster‐level FWE correction *p* <  .05, cluster size > 30.

**FIGURE 4:**
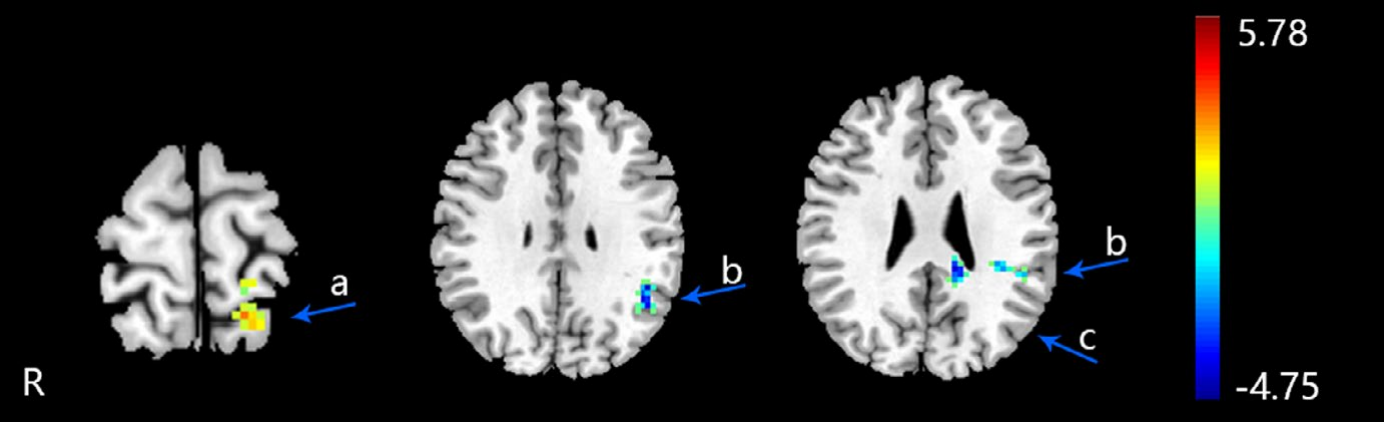
The differences of altered fALFF regions between MPIs and FPIs. (an uncorrected voxel‐wise *p*  <  .005 and cluster‐level FWE correction *p* <  .05, cluster size > 30). R: right brain. a: left postcentral gyrus, b: left supramarginal gyrus, c: left posterior cingulate

There were differences between the altered fALFF regions of MPIs and the altered fALFF regions of FPIs, which were mainly in the left posterior cingulate, left supramarginal gyrus, and left postcentral gyrus. The fALFF values of the left supramarginal gyrus and left posterior cingulate were increased in FPIs, while no change in these regions was found from MPIs. The fALFF in left postcentral gyrus was increased in MPIs, while no change in this region was found from FPIs (an uncorrected voxel‐wise *p*  <  .005 and cluster‐level FWE correction *p* <  .05, cluster size > 30; Table 5 and Figure 4).

**TABLE 5:** Difference of altered fALFF regions between MPIs and FPIs after 5‐weeks EA treatment at HT‐7

Brain regions	BA	Side	Cluster size	MNI			*t*‐value
				*X*	*Y*	*Z*	
Posterior cingulate		L	42	−12	−39	21	−4.75
Supramarginal gyrus	39	L	38	−48	−48	30	−4.55
Postcentral gyrus		L	32	−18	−48	75	5.58

Note

Anatomical locations, approximate Brodmann areas (BA), and Montreal Neurological Institute (MNI) coordinates, correspond to the *t*‐values of representative peaks within each cluster were reported. L: left. R: right. A positive *t*‐value means that altered fALFF in this brain region of MPIs was increased higher than that in FPIs, while a negative *t*‐value means that fALFF in this brain region of FPIs was increased higher than MPIs. All regions reached an uncorrected voxel‐wise *p*  <  .005 and cluster‐level FWE correction *p* <  .05, cluster size > 30.

## 4 DISCUSSION

The AIS and PSQI scores in MPIs and FPIs significantly decreased after the 5‐week EA treatment, suggesting EA treatment was effective in reducing the symptoms of patients and improving patients' sleep experience. This result is consistent with the findings of other research about acupuncture for insomnia (Yin et al., 2017; Zhao, 2013). The rs‐fMRI results suggested that the spontaneous brain activity of male PI patients and female PI patients changed after 5 weeks of EA treatment. In the MPIs group, the fALFF increased in the left middle temporal gyrus, left superior frontal gyrus, left middle frontal gyrus, left medial frontal gyrus, right precuneus, right parahippocampal gyrus, left superior parietal lobule, and bilateral postcentral gyrus. In the FPIs group, the fALFF increased in the left middle temporal gyrus, left superior temporal gyrus, left inferior frontal gyrus, left posterior cingulate, left middle frontal gyrus, and left supramarginal gyrus.

The frontal lobe has always been playing an important role in brain functions such as emotion and cognition. The middle frontal gyrus as one of the major nodes of the executive control network is involved in executive functions broadly, such as attention, working memory, and episodic memory (Kay et al., 2016). The medial frontal lobe is considered to subserve emotion and personality (Smith et al., 2017). In previous research, the deactivation in medial frontal gyrus was found in bipolar disorder patients when compared to healthy participants (Townsend et al., 2013). The inferior frontal gyrus has been implicated in inhibitory processes in numerous cognitive and emotional paradigms (Shafritz et al., 2006). Research has suggested that this region might be involved in the suppression or regulation of anxiety (Monk et al., 2006). The precuneus is involved in diverse processes, such as attention, episodic memory retrieval, working memory, and conscious perception. These provide the rich episodic contextual associations used by the prefrontal cortex to select correct past memory attention (Zhang & Li, 2012). The precuneus is significantly deactivated during rapid eye movement (REM) sleep, which may be responsible for reduced attention and cognitive control, illogical thinking, and impaired working memory in the dream experience (Perogamvros & Schwartz, 2015). Some studies have shown that the activity of precuneus is different between PI and healthy people (Dai et al., 2016). Parahippocampal gyrus and cingulate gyrus are critical memory‐related structures, which are related to the emotional and cognitive functions. Parahippocampal gyrus is thought to be involved in the intersection between perception and memory, as well as the translation of material into more permanent storage in the cortical association areas (Yogarajah et al., 2008). The research has found reduced activation in parahippocampal gyrus following sleep deprivation (Ma et al., 2015). Cingulate gyrus, an integral part of the limbic system, is involved with emotion formation and processing, learning and memory (Kozlovskiy et al., 2012) and the sleep process (Murphy et al., 2009). Posterior cingulate cortex (PCC) may play a direct role in regulating the focus of attention; its activity varies with arousal state and its interactions with other brain networks may be important for conscious awareness (Leech & Sharp, 2014; Vogt & Laureys, 2005). Moreover, middle temporal gyrus and supramarginal gyrus are the regions associated with verbal divergent thinking, which can be activated during a divergent thinking task (Cousijn et al., 2014). Divergent thinking involves the ability to generate novel and useful problem solutions, which is an important component of creativity (Cousijn et al., 2014).

Insomnia is usually associated with emotional disorders, and the excitable increase in emotion is an important factor in the etiology of insomnia (Riemann et al., 2010). The emotional network is a necessary factor for the emergence and maintenance of consciousness in a developing brain, which is maintained through the sleeping process. Sleep disturbances may lead to emotional and cognitive dysfunctions (Medic et al., 2017) and versa vice (Baglioni et al., 2016). Therefore, we speculated that changes in spontaneous brain activity in the brain regions associated with emotional and cognitive regulation might be the potential mechanism of EA to play a therapeutic role.

However, our clinical results suggested the decreased PSQI score in FPIs was significantly higher than that of MPIs (*p* < .05). Although there was no significant difference in the decreased AIS score between MPIs and FPIs, the decreased AIS score in FPIs was higher than that in MPIs. These findings suggested that there might be gender differences in the treatment efficacy of EA, and female patients might benefit more from EA than male patients. Gender‐related differences in efficacy have also been found in studies of acupuncture for other diseases such as depression (Fan et al., 2015).

We considered that one of the reasons for the gender‐related difference of efficacy might be the gender‐related difference in the cerebral response to acupuncture, which was mainly in PCC and supramarginal gyrus. Previous researches have detected brain activity during the acupuncture intervention. The results found that acupuncture stimulation worked differently in the brain between males and females (Qiu et al., 2010; Yeo et al., 2016).

The gender‐related difference in the cerebral response to acupuncture is due to the different spontaneous brain activity between males and females. In our rs‐fMRI study, gender‐related differences of spontaneous brain activity were found in the bilateral middle temporal gyrus, right cuneus, left supramarginal gyrus, left precuneus, left posterior cingulate, bilateral inferior parietal lobule, and bilateral cingulate gyrus. Specifically, the fALFF values of these regions in MPIs were higher than those in FPIs. Previous studies have reported gender differences in functional brain networks (Xu et al., 2015), cognitive regulation (Kogler et al., 2015), and neural activity during emotional regulation (Mak et al., 2009). Consistent with these findings, our study indicated that gender differences might affect fMRI signals and outcomes induced by acupuncture. The gender factor should be considered in further research on the mechanism of acupuncture.

## 5 LIMITATION

This study only discusses the results of single acupoint HT‐7 that is widely used in clinical treatment for insomnia, but still need to explore whether there are similar conclusions in other acupoints used in the clinic. This should be explored in further research.

## CONFLICT OF INTEREST

The authors declare no competing financial interests.

## AUTHOR CONTRIBUTION

All authors were involved in the conception and design of the analyses. Xiao‐Hua Shi performed the clinical experimental work. Tie Li, Yu‐Kai Wang, Hong‐Yu Liu, Xin‐Tong Wang, and Zhi‐Hong Wang had contributions to data collection. Xiao‐Hua Shi and Yu‐Kai Wang wrote the manuscript and did the fMRI data analysis. Professor Jing Mang and Zhong‐Xin Xu provided decisive feedback and suggestions to improve the manuscript.

### Peer Review

The peer review history for this article is available at https://publons.com/publon/10.1002/brb3.1927.

## Data Availability

The datasets generated and/or analyzed in the present study can be available from the corresponding author upon reasonable request.
